# The King Edward VII. Sanatorium

**Published:** 1903-11-07

**Authors:** 


### THE KING EDWARD VII. SANATORIUM.

The King laid the foundation-stone of his Sanatorium for Tuberculosis at Lord's Common, near Midhurst, on Tuesday. His Majesty travelled from Waterloo Station to Haslemere and drove thence to Lord's Common, where a large company had assembled.

The preparations were of an elaborate character. A pavilion, consisting of a set of waiting-rooms, had been erected by the side of the new road which has been driven through the pine woods to the site of the sanatorium. Beyond this pavilion lay the level space, surrounded by woods and commanding a fine distant view, where the buildings will stand. Here the ceremony of laying the foundation-stone was arranged to take place. In the centre was a raised and covered platform, above which the stone itself hung poised. A semicircle of covered seats held the invited guests, to the number of some 600, on one side; and on the other side, where the ground broke away, was a semicircle of uninvited spectators. On the arrival of the King, Sir William Broadbent read an address in which he pointed out the numerous advantages of the site and explained the principles upon which the planning of the sanatorium had been based. He mentioned that the total number of beds would be 100, of which 12 would be reserved for well-to-do patients.

In replying to the address the King expressed his satisfaction at the site selected by the Committee, and thanked them for their assistance. He alluded with regret to the Queen's inability to accompany him on the occasion, as she was much interested in the fight against tuberculosis. The King concluded by expressing his hope that the Sanatorium would be the means of restoring to health many who came there. Before leaving, His Majesty spent some time in studying the plans and drawings of the future building with Mr. Percy Adams, the architect. It is hoped that the building will be completed in a year's time.

